# Reproducibility of diffusion, shape, and connectivity metrics across scanners: Implications for multi-site tractography

**DOI:** 10.1016/j.neuroimage.2026.122228

**Published:** 2026-09-11

**Authors:** Sachit Anand, Fang-Cheng Yeh, Siva Venkadesh

**Affiliations:** a Department of Neuroscience, University of Pittsburgh, Pittsburgh, PA, USA; b Department of Computer Science, University of Pittsburgh, Pittsburgh, PA, USA; c Department of Neurological Surgery, University of Pittsburgh, Pittsburgh, PA, USA

**Keywords:** Diffusion MRI, Tractography, Reproducibility, Harmonization, Multi-site, Structural connectivity, Tract morphometry

## Abstract

Multi-site diffusion MRI studies face scanner-induced variability that can obscure biological signal. Harmonization methods such as ComBat have been developed to address this, but have been evaluated primarily on diffusion scalar metrics. Whether scanner reproducibility differs across fundamentally distinct tract-derived representations has not been systematically compared.

Here, we compared the reproducibility of three metric families (diffusion, shape, and connectivity) across 36 association tracts using the MASiVar dataset (5 subjects, 4 scanners, 27 sessions). We assessed intraclass correlation coefficients (ICC) and multivariate subject discrimination at baseline, under dimensionality reduction, and after ComBat harmonization.

At baseline, shape metrics showed the highest scanner reproducibility (median ICC 0.69), followed by connectivity (0.49) and diffusion (0.34). Shape and connectivity achieved comparable subject discrimination (both 1.75), significantly exceeding diffusion (1.23). ComBat harmonization improved all families but harmonized diffusion (0.58) remained below unharmonized shape (0.69), indicating that metric family selection remains consequential even after harmonization.

Under low-dimensional representation, connectivity showed the largest gains (ICC 0.86, subject discrimination 3.0), exceeding other families at any dimensionality. Analysis of principal component loadings identified a small number of cortical regions per tract (median 6) that capture 95% of the reproducible connectivity signal, providing a per-tract reference for selecting the most informative regions in future multi-site studies.

These findings indicate that the choice of which tract-derived metrics to analyze in multi-site studies deserves at least as much consideration as how to harmonize them.

## 1. Introduction

Multi-site diffusion MRI studies increasingly rely on tract-derived measures to characterize white matter organization, yet the reproducibility and scanner sensitivity of commonly used tract representations remain incompletely understood. Prior work has demonstrated that diffusion scalars, tractography-based connectivity measures, and geometric descriptors can each exhibit substantial scanner effects, and that harmonization methods such as ComBat (Fortin et al., 2017) and RISH (Mirzaalian et al., 2016) may mitigate these effects.

However, existing evaluations of scanner variability, including large-scale harmonization efforts that have successfully established normative reference curves for diffusion scalars across tens of thousands of subjects (Cetin-Karayumak et al., 2024; Zhu et al., 2025, 2019), have focused primarily on metrics within a single family, typically FA and MD. Recent work has begun addressing connectivity directly, with (Kurokawa et al., 2021) demonstrating that site effects in structural connectomes persist after signal-level harmonization and require correction at the matrix level, and (Onicas et al., 2022) applying ComBat to connectome-derived graph metrics. However, a systematic comparison of scanner reproducibility across fundamentally different tract representations (diffusion microstructure, tract geometry, and regional connectivity) has not been undertaken, nor has prior work examined whether multivariate subject discrimination (Finn et al., 2015), which captures the joint signal across features, reveals reproducibility structure invisible to univariate measures such as the intraclass correlation coefficient (ICC) (Shrout and Fleiss, 1979). This matters because different metric families occupy different positions in the processing hierarchy: diffusion metrics summarize voxel-level microstructure, shape metrics capture tract-level geometry, and connectivity metrics describe region-level overlap patterns. These successive levels of spatial abstraction may confer different degrees of inherent robustness to scanner variability.

We address this gap by decomposing the total variability in each tract-derived metric into between-subject and within-subject components. By comparing the three metric families under both native and dimension-matched (PCA) representations, and before and after ComBat harmonization, our results offer practical guidance for selecting tract-derived metrics in multi-site studies and clarify how representation dimensionality shapes reproducibility across scanners.

## 2. Methods

### 2.1. Dataset

MASiVar Cohort II (Cai et al., 2021) comprises 27 diffusion MRI sessions acquired from 5 healthy adults across 4 scanners at 3 sites. Each session corresponds to a single scan acquisition of one subject on one scanner. Preprocessed data were downloaded from OpenNeuro (ds003416), where all scans had been processed with PreQual v1.0.0, including Marchenko-Pastur denoising, intensity normalization, susceptibility and eddy current distortion correction, motion correction, and slice-wise signal dropout imputation.

#### Ethics statement.

As reported by Cai et al. (2021), all original scans were acquired only after informed consent under the supervision of the project Institutional Review Board. The present study is a secondary analysis of these de-identified, publicly available data and involved no new data collection or interaction with human participants. It was conducted under the University of Pittsburgh Institutional Review Board protocol STUDY19040351 (approved June 28, 2019), which covers image analysis of publicly available imaging data.

### 2.2. Pipeline

Diffusion data were reconstructed using DSI Studio (Yeh, 2021) (version “Hou” Aug 2024) with generalized q-sampling imaging (GQI, diffusion sampling length ratio = 1.25) and resampled to 2.5 mm isotropic resolution. Volumes were rigidly aligned to AC-PC orientation prior to reconstruction. Thirty-six bilateral association tracts were identified using automatic tract recognition, which matches subject streamlines to a population-based tractography atlas via nonlinear registration and Hausdorff distance filtering.

For each tract, three families of metrics were extracted (Fig. 1). Shape metrics (19 per tract) were derived from the geometric properties of reconstructed streamlines (Yeh, 2020), including tract count, mean length, span, curl, elongation, total volume, quarter-segment volumes, surface area, irregularity, and endpoint area, radius, and branch volume for each terminus. Definitions and units for all shape and connectivity metrics are provided in Supplementary Table S1. Diffusion metrics (13 per tract) were computed as mean values along each tract's streamlines, comprising DTI-derived scalars (FA, MD, AD, RD), GQI-derived measures (QA, GFA, ISO, RDI, HA), and restricted diffusion indices (RD1, RD2, nRDI at b = 2000 and b = 4000).

Connectivity metrics were derived from tract-to-region overlap analysis. Quantifying the spatial overlap between axonal pathways and parcellated regions has a long history in both tractography (Donahue et al., 2016; Hagmann et al., 2008; Owen et al., 2015) and viral tracing connectomics (Oh et al., 2014). (Yeh, 2022) defined a population-level tract-to-region connectome that quantifies connectivity for each tract independently; here, we extend it to individual subjects. For each tract, a track density image (TDI) is computed by rasterizing all streamlines onto the native diffusion voxel grid, counting distinct streamlines per voxel. The FreeSurferSeg parcellation, comprising the Destrieux cortical atlas (Destrieux et al., 2010) together with FreeSurfer subcortical segmentation (Fischl et al., 2002), for a total of 192 regions, is warped nonlinearly to subject space, and for each region, the intersect ratio is computed as the fraction of region voxels where tract density exceeds a noise floor (0.5% of maximum TDI value). This yields a value between 0 and 1 per tract–region pair, representing the spatial overlap between each tract and each cortical or subcortical region. The number of regions with nonzero overlap varies per tract (range: 12–56), reflecting each tract's cortical territory.

### 2.3. Reproducibility metrics

We characterize the reproducibility of each metric across scanners, by which we mean the degree to which the same metric value is recovered when a subject is scanned on a different scanner. We assess this at two complementary levels.

#### Intraclass Correlation Coefficient (univariate):

We computed ICC (1,1) (Shrout and Fleiss, 1979), a reliability coefficient, from a one-way random-effects model, which quantifies absolute agreement and treats a subject's repeated sessions as exchangeable replicates. This model does not partition within-subject variance into separate components; all non-subject variance is pooled into the denominator, so the ICC reflects agreement across sessions regardless of the source of variation. Unequal numbers of sessions across subjects were accommodated using the harmonic mean of the group sizes, which keeps the estimator well defined and bounded at zero when between-subject variance is absent.


(1) $$ICC=\frac{\sigma_{between}^{2}}{\sigma_{between}^{2}+\sigma_{within}^{2}}$$

Here, $\sigma_{between}^{2}$ reflects biological differences across subjects and $\sigma_{within}^{2}$ reflects within-subject variance across repeated sessions on different scanners.

#### Subject discrimination (multivariate):

Rather than evaluating metrics independently, subject discrimination assesses whether the full profile of metrics within a family jointly distinguishes individuals across scanners. It is the multivariate analogue of the ICC: where ICC compares between- and within-subject variance for a single metric, subject discrimination compares between- and within-subject distances over the full feature set, quantifying subject-level signal relative to scanner-induced variation. The principle that individuals are distinguishable from their imaging profiles is established in previous connectome fingerprinting work (Finn et al., 2015); our measure operationalizes it as a between-subject to within-subject distance ratio. For shape and diffusion, features are z-score (centered to zero mean and scaled to unit standard deviation, computed per feature across all sessions) normalized prior to distance computation to account for heterogeneous measurement scales; connectivity features, which are uniformly expressed as intersect ratios, are used without standardization to preserve the natural variance structure.


(2) $$Subject\;discrimination=\frac{med(D_{between})}{med(D_{within})}$$

Here, med denotes median, $D_{between}$ is the set of pairwise Euclidean distances between sessions from different subjects, and $D_{within}$ is the set of pairwise Euclidean distances between sessions from the same subject (across all of that subject's sessions, spanning scanners and repeated acquisitions). Both are computed over all metrics in the family jointly. A value of 1 indicates no subject separability; higher values indicate that between-subject distances exceed within-subject distances.

In all comparisons, the tract is the unit of analysis, with metric families compared within each of the 36 association tracts. Although these tracts derive from the same subjects, each is recognized independently through the automated tractography pipeline (Yeh, 2020).

### 2.4. Dimensionality analysis (PCA)

The three metric families differ substantially in native dimensionality: shape contributes 19 features per tract, diffusion 13, and connectivity 12–56 depending on the tract's cortical extent. To enable fair cross-family comparison and to assess how reproducibility across scanners depends on representational dimensionality, principal component analysis (PCA) was applied independently within each metric family for each tract. For shape and diffusion, features were standardized prior to PCA to account for heterogeneous scales (correlation-based PCA); for connectivity, PCA was applied to the raw covariance matrix to preserve the natural variance hierarchy among intersect ratios. ICC and subject discrimination were recomputed at each dimensionality from 1 to 20 retained components. This analysis reveals whether reproducible subject-specific signal is concentrated in a few dominant modes or distributed across the full feature space, a distinction that proves critical for connectivity metrics in particular. The stability of the connectivity loadings under leave-one-scanner-out and bootstrap resampling was assessed as described in Supplementary Material.

### 2.5. Harmonization (ComBat)

To evaluate the effect of scanner harmonization on reproducibility, ComBat (Fortin et al., 2017) was applied to the baseline (non-PCA) features within each metric family. Scanner identity served as the batch variable, and subject identity was included as a biological covariate to preserve inter-individual differences during harmonization. ICC was recomputed after harmonization to quantify the gain attributable to removing scanner-specific variance.

As a sensitivity analysis, we additionally applied ComBat with scanner as the only batch variable and no subject covariate, corresponding to a conventional multi-site setting in which the same individuals are not scanned at every site.

## 3. Results

### 3.1. Baseline metric family comparison

Fig. 2 illustrates the raw variability across scanners and subjects for a representative tract (left arcuate fasciculus), showing how within-subject and between-subject differences co-exist in the raw metric values.

At baseline, shape metrics showed the highest reproducibility across the 36 association tracts (Fig. 3), with a median ICC of 0.69, followed by connectivity (0.49) and diffusion (0.34). All pairwise differences were significant (Shape > Diffusion, p 〈 0.001; Shape 〉 Connectivity, p 〈 0.001; Connectivity 〉 Diffusion, p < 0.01). For subject discrimination, shape and connectivity were statistically indistinguishable (medians 1.75 and 1.75, respectively; p = 1.000), while both significantly exceeded diffusion (1.23; p < 0.001). Shape thus leads in per-metric reproducibility, but shape and connectivity are equally effective at distinguishing individuals when the full feature profile is considered. Diffusion is lowest on both measures.

ICC varied substantially across tracts within each family (Supplementary Figure S1). Longer association tracts with distinctive geometry, such as PAT-R (0.88), SLF2-R (0.86), ILF-L (0.83), and ILF-R (0.82), showed the highest shape reproducibility, likely because their size and trajectory make them reliably identifiable across scanners. The lowest-performing tracts were predominantly cingulum subdivisions, including CgL-PHP (0.28), CgL-pH (0.39), CgL-FPH (0.46), and CgL-SLF1 (0.47), which are shorter and more sensitive to registration variability. Connectivity ICC showed the most tract-to-tract variability, with some tracts matching or exceeding shape (e.g., PAT-L connectivity 0.81) while others fell well below diffusion (e.g., HA-L connectivity 0.23, EC-L connectivity 0.22), reflecting dependence on the number and distinctiveness of cortical overlap regions per tract.

### 3.2. Effect of dimensionality reduction

To assess how reproducibility depends on representational dimensionality, we applied PCA within each metric family separately for each tract and recomputed ICC and subject discrimination as a function of the number of retained components (Section 2.4). At one component, all pairwise family differences were significant (paired Wilcoxon, p < 0.001 each; Fig. 4A-B). Connectivity ICC reached approximately 0.86, far above its baseline of 0.49. Shape ICC rose to approximately 0.75 (from 0.69), while diffusion showed a more modest increase to approximately 0.48 (from 0.34). Subject discrimination followed a similar pattern: connectivity reached approximately 3.0 at one component, well above its baseline of 1.75. Shape rose to approximately 2.4 and diffusion to approximately 1.4 at two components. At 1–3 components, connectivity ICC and subject discrimination exceeded all other families at any dimensionality. For subject discrimination, all families converged to their respective baseline values at high component counts, confirming that full-rank PCA recovers the native feature space. ICC does not show this convergence because it is a per-component property rather than a joint distance measure.

All three families showed improved reproducibility at low component counts, but connectivity benefited disproportionately due to its larger and more correlated feature space (see Discussion). Because PCA is fit per tract, the regions driving the first component differ across tracts. Inspection of PC1 loadings confirms that the highest-loading regions correspond to known termination territories for each tract (e.g., amygdala for hippocampal-amygdala tracts, insular cortex for extreme capsule, intraparietal sulcus for SLF; Supplementary Table S2). Retaining regions that account for 95% of PC1 loading variance selects a median of 6 regions per tract (range 2–14), adapting to each tract's loading concentration. Mapping these regions onto cortical surface across all 36 tracts shows that 126 of 192 FreeSurferSeg regions contribute to reproducible individual variation in connectivity (Fig. 4C). Resampling confirmed that the connectivity PC1 loadings and their top-weighted regions are stable across scanners (median Tucker's congruence coefficient (Lorenzo-Seva and ten Berge, 2006) 0.99 leave-one-scanner-out and 0.96 bootstrap; 95%-loading region recall 0.95 and 0.90; Supplementary Material, Supplementary Figure S2, Supplementary Table S3).

### 3.3. Effect of harmonization

ComBat harmonization, applied separately within each metric family, improved ICC in every family (all 36 tracts improved in each family; paired Wilcoxon, p < 10^−6^; Fig. 5). Shape increased from 0.69 to 0.77 (+12%), diffusion from 0.34 to 0.58 (+72%), and connectivity from 0.49 to 0.62 (+27%). Despite these gains, the cross-family hierarchy persisted. To test whether harmonization can substitute for metric choice, that is, whether correcting diffusion for scanner effects brings it up to the baseline reliability of a more scanner-robust family, we compared harmonized diffusion with unharmonized shape. Harmonized diffusion (0.58) remained below unharmonized shape (0.69; rank-biserial correlation *r_rb_* = −0.41), indicating that harmonization does not close the gap between families. Metric family selection therefore remains consequential after harmonization, with implications for statistical power and required sample size in multi-site studies (see Section 4.3).

To confirm that this result does not depend on the inclusion of subject identity as a covariate, which may yield optimistic reliability estimates relative to a conventional multi-site setting, we repeated the harmonization using scanner as the only batch variable. The cross-family ordering was unchanged: harmonized diffusion (median ICC 0.53) remained below unharmonized shape (0.69), and the effect size increased slightly (*r_rb_* = −0.66; Supplementary Table S4, Supplementary Figure S3).

It is worth noting that for connectivity, dimensionality reduction (ICC 0.86 at one component) produced larger gains than ComBat harmonization (ICC 0.62), whereas for shape and diffusion the two approaches yielded comparable improvements. This pattern suggests that for connectivity, appropriate representation of the distributed biological signal yields larger reproducibility gains than scanner correction alone.

## 4. Discussion

Diffusion MRI tractography has become a central tool for investigating white matter organization across clinical and developmental applications (Assaf et al., 2019). As multi-site consortia scale these studies, the choice of which tract-derived measures to analyze becomes consequential, yet is rarely treated as a methodological variable. In practice, many studies prioritize diffusion tensor–derived scalars such as FA and MD, which are among the most established metrics in the literature (Figley et al., 2021; Jones et al., 2013; Nir et al., 2017). This prevalence is understandable: DTI scalars are straightforward to compute and have accumulated extensive normative data, and the field's harmonization infrastructure has developed primarily around them (Cetin-Karayumak et al., 2024; Zhu et al., 2025, 2019). Our results suggest, however, that this concentration may not reflect an optimal allocation of effort: diffusion metrics were the least reliable across scanners among the three families in this dataset, and this disadvantage persisted even after harmonization. We describe these findings as reproducibility across scanners, following standard usage in the multi-site harmonization literature (Kurokawa et al., 2021), since our measures quantify the degree to which the same values are recovered when a subject is scanned on a different scanner.

### 4.1. Why shape metrics lead at baseline

Shape metrics describe macroscopic tract geometry derived from reconstructed streamlines. Each step in the processing chain, from voxel-level signal to fiber orientation to streamline trajectory to geometric summary, integrates over progressively larger spatial scales and reduces sensitivity to voxel-level noise and some scanner-dependent variability. Properties such as volume, length, and curvature reflect aggregate characteristics of many (correlated) streamlines rather than signal intensity at any individual voxel, making them less sensitive to differences in gradient strength, noise floor, or signal-to-noise ratio. While connectivity overlap is also derived from streamline trajectories, it remains more sensitive to endpoint definitions and spatial alignment, which may limit the extent of this robustness in individual connections. This geometric stability also underlies shape's advantage in ICC: individual differences in tract anatomy, such as the size and curvature of major association pathways, are well captured by shape descriptors, exhibit between-subject variability that exceeds measurement noise, and remain relatively consistent across scanning conditions. Notably, connectivity achieves comparable subject discrimination despite lower per-metric ICC, suggesting that its biological signal is distributed across many features rather than concentrated in individual metrics.

### 4.2. Why connectivity uniquely benefits from PCA

All three metric families contain internally correlated features: diffusion scalars covary through the shared tensor, shape metrics scale together with tract size, and connectivity overlap values reflect shared spatial patterns. PCA exploits this redundancy in each family, explaining why even shape and diffusion ICC improve at low component counts. Connectivity benefits disproportionately, however, because its subject-specific signal is distributed across many small, consistent regional differences that aggregate into a strong first component. The result is a representation where 1–3 components per tract capture nearly all reproducible variance, an effective subject fingerprint inaccessible from any single connectivity value.

Examination of PC1 loadings supports the anatomical validity of this compression. For each tract, the highest-loading regions correspond to expected termination zones, and the top 2–3 regions account for a median of 82% of PC1 variance (Supplementary Table S2). In roughly half of tracts (19/36), the top loadings include both positive and negative signs (Fig. 4D), indicating that the primary axis of individual variation is not overall tract size but rather a trade-off between termination regions. For example, in the left arcuate fasciculus, posterior temporal-occipital regions load positively while the planum temporale loads negatively, capturing known individual variation in where the arcuate terminates along the temporal lobe. These mixed-sign loadings imply that reproducible individual differences often take the form of trade-offs between termination regions, information that would be lost under univariate analysis of individual region overlaps.

### 4.3. Practical implications for multi-site study design

Our results do not establish that any one metric family is biologically superior. Shape, diffusion, and connectivity metrics capture complementary aspects of white matter organization: macroscopic tract geometry, tissue microstructure, and regional structural connectivity, respectively. Greater reproducibility across scanners does not confer greater biological validity, and the established status of diffusion scalars does not make them more valid than the less familiar shape and connectivity measures. Our recommendation is therefore conditional rather than a ranking. When a scientific question can be addressed through tract geometry or regional connectivity, these representations offer greater robustness to scanner variation. The reproducibility cost of relying on diffusion scalars is concrete: because their higher scanner-induced variance persists after harmonization, it inflates within-subject measurement noise, lowering statistical power and increasing the sample size required to detect a given biological effect in a multi-site study. This cost should be weighed against the microstructural specificity that diffusion scalars provide.

For connectivity, not all regions contribute equally to reproducible individual signal. The per-tract loading structure reported here (Fig. 4D, Supplementary Table S2) identifies which regions carry the most reliable between-subject variation, and these regions should be analyzed jointly to preserve the trade-off structure revealed by mixed-sign loadings. For studies using different pipelines or parcellations, a covariance-PCA on a small reference subset can identify the equivalent high-loading regions. This recommendation applies to study designs where individual differences are the target (e.g., group comparisons, biomarker development). For applications requiring the full connectivity representation, such as systems-level network modeling, population-stable connections with near-zero PC1 loadings remain essential as the structural scaffold of the circuit.

More broadly, multi-site studies may benefit from considering whether their scientific question requires voxel-level microstructural measures, or whether it can be addressed using higher-level derived quantities that carry less scanner-induced variance. Diffusion scalars remain essential when microstructural specificity is the object of study, but our results suggest that scanner-robust alternatives exist and may be preferable when the scientific question permits.

### 4.4. Limitations

The sample comprises 5 subjects across 4 scanners. Because each subject is scanned on multiple scanners, between-subject and within-subject variance can be separated, which is sufficient for the reliability measures reported here (ICC and subject discrimination). The within-subject term reflects variation across sessions and scanners rather than scanner differences alone; we minimized other potential contributors by analyzing data preprocessed through a single uniform pipeline (PreQual, including motion correction) and by recognizing every tract through the same automated pipeline, so that analytic sources such as preprocessing and tract-recognition variability are held essentially constant across scans. Estimating scanner sensitivity as a distinct quantity would require multiple sessions within the same scanner per subject, which was available for only two of four scanners, precluding reliable quantification. Future work could extend this framework using larger traveling-subject designs where within-scanner replication is available.

Additionally, all analyses were conducted using a single tractography platform. While this could limit generalizability, it also ensures that cross-family comparisons are not confounded by differences between processing tools, as the platform used is one of few that provides tract-level shape, diffusion, and connectivity metrics within a unified pipeline. Because several tracts connect to more cortical regions than there are scans, individual region loadings beyond the dominant set are estimated with limited precision; we therefore base our conclusions on the leading low-dimensional component and its top-weighted regions, whose cross-scanner stability we verify directly (Supplementary Material).

Our statistical comparisons treat the tract as the unit of analysis. Because all tracts derive from the same five subjects, scanners, and pipeline, tract-level values are not fully independent, and the reported p-values are best read as describing the consistency of family-level differences across tracts rather than as inference to a broader population. We note, however, that each tract is recognized independently and that the per-tract reliability profiles of the three families are not systematically coupled (Supplementary Figure S1): a tract with high shape reliability does not predictably show high diffusion or connectivity reliability. This suggests that the family-level differences we report are driven by tract anatomy rather than by the small number of shared participants.

Our findings are bounded by the dataset and pipeline used: five healthy adults, a single tractography platform, one parcellation and tract-to-region overlap strategy, and a specific set of 36 association tracts. The specific reliability values and the magnitude of the differences between metric families should therefore be read as specific to this MASiVar/DSI Studio framework. The qualitative ordering we observe follows from a general mechanism, that representations integrating over larger spatial scales are less sensitive to scanner-dependent signal variation, and we expect it to hold more broadly, but its quantitative expression may differ under other platforms, parcellations, or populations, and confirming this is a direction for future work.

## 5. Conclusion

In the MASiVar traveling-subject dataset, shape metrics achieved the highest baseline reproducibility across scanners, and connectivity metrics, under simple dimensionality reduction, yielded the strongest subject-specific signatures. ComBat harmonization improves all families but does not eliminate the scanner reproducibility hierarchy. These findings suggest that the question of which metrics to use in multi-site studies deserves at least as much attention as the question of how to harmonize them.

## Supplementary Material

1

2

3

Supplementary material associated with this article can be found, in the online version, at doi:10.1016/j.neuroimage.2026.122228.

## Figures and Tables

**Fig. 1.**
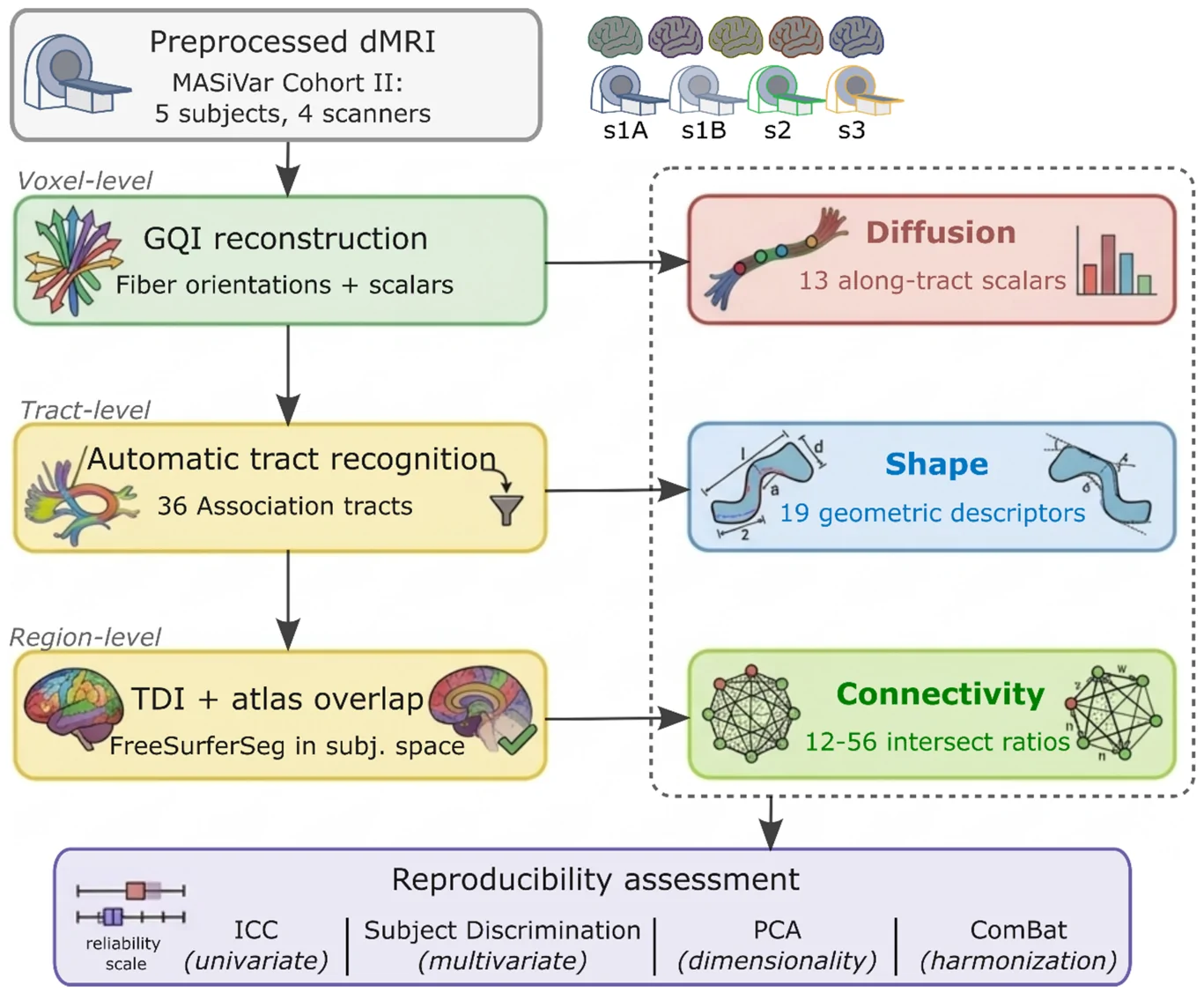
Processing pipeline and metric family overview. Preprocessed diffusion MRI data from MASiVar Cohort II (5 subjects, 4 scanners at 3 sites; scanner icons colored by site) are processed through successive stages yielding three metric families at different levels of spatial abstraction: diffusion (voxel-level), shape (tract-level), and connectivity (region-level). All three families are evaluated using four reproducibility assessments (bottom).

**Fig. 2.**
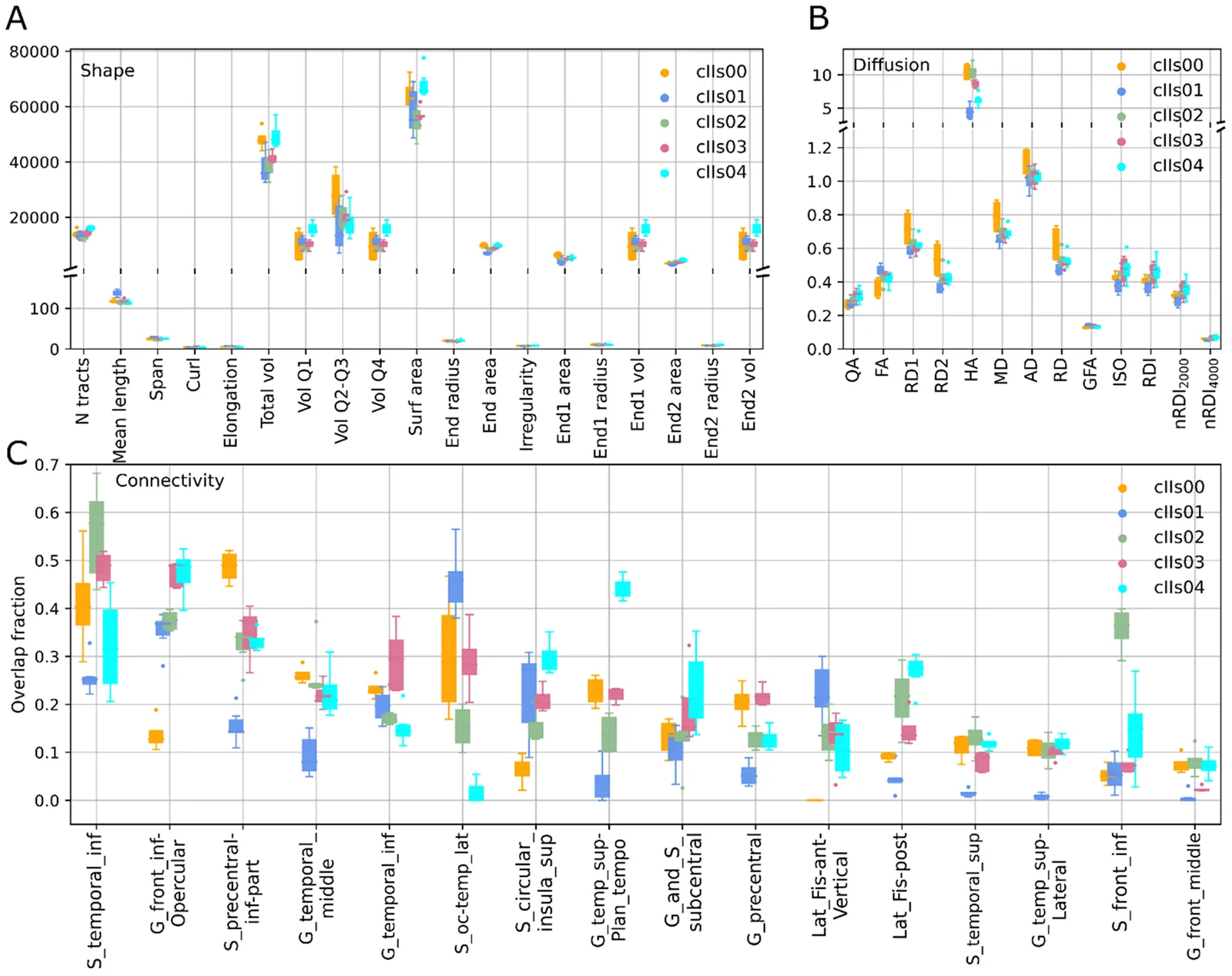
Raw variability of tract-derived metrics across scanners and subjects for the left arcuate fasciculus. Each panel shows the distribution of metric values across the scanning sessions of each subject, with the five subjects distinguished by color. Subject labels denote MASiVar Cohort II subjects (cII, Cohort II; s, subject; e.g., cIIs00). Within each metric, box plots show the median (center line), interquartile range (box), and whiskers (1.5 × IQR). (A) Shape metrics (19 per tract); the y-axis is broken to accommodate the differing scales of count, length, area, and volume measures, and metric abbreviations are defined in Supplementary Table S1. (B) Diffusion metrics (13 per tract), labelled with standard diffusion MRI abbreviations as defined in Section 2.2. (C) Connectivity metrics for the regions with the highest overlap with this tract; values are intersect ratios (0–1) and region labels follow the Destrieux atlas nomenclature (Destrieux et al., 2010).

**Fig. 3.**
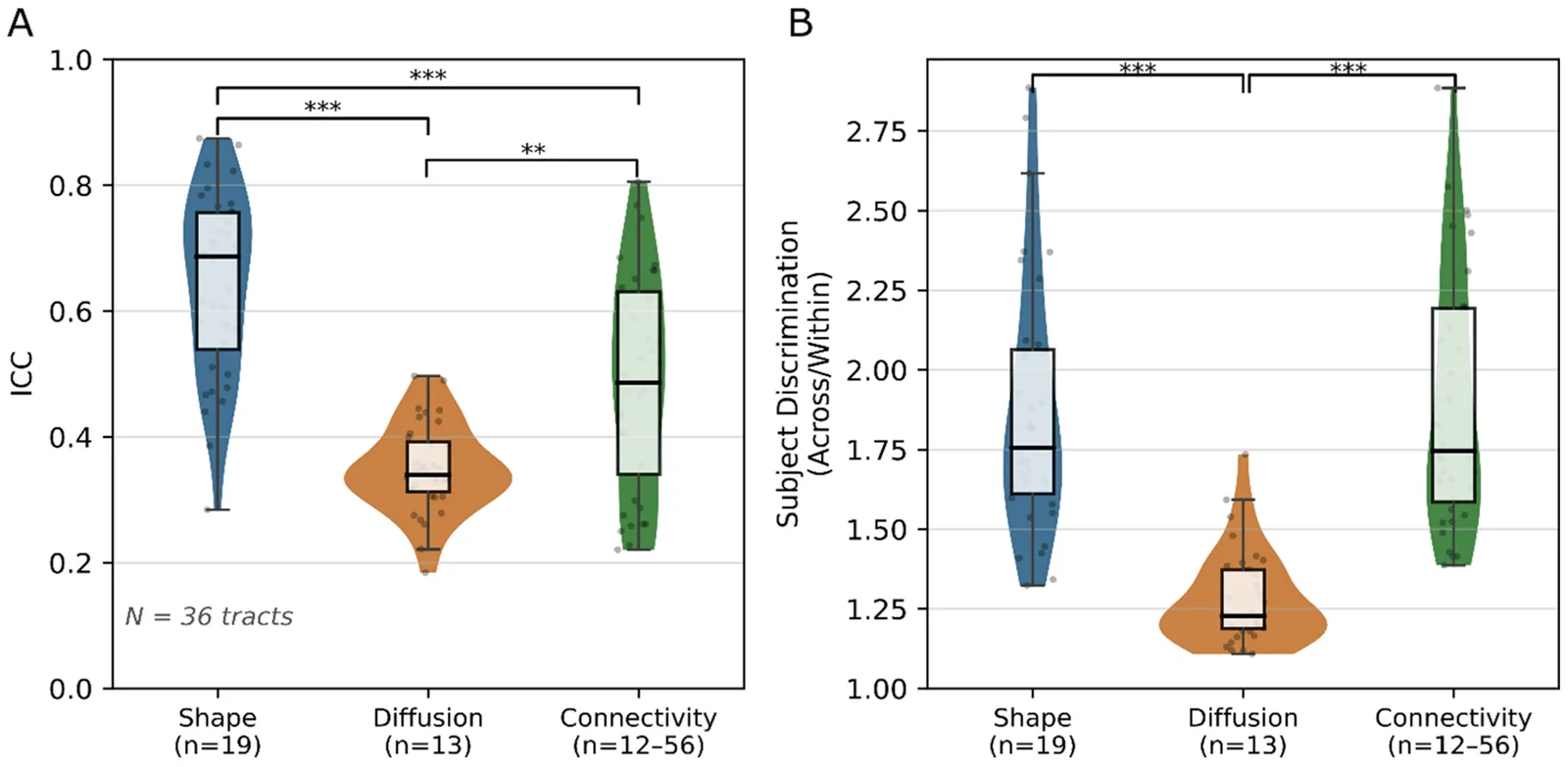
Baseline scanner reproducibility and subject discrimination across metric families. (A) Violin plots of tract-level ICC (a reliability coefficient quantifying agreement across scanners) for shape, diffusion, and connectivity across 36 association tracts. Significance brackets indicate pairwise Wilcoxon signed-rank tests. (B) Multivariate subject discrimination (ratio of across-subject to within-subject Euclidean distance) for each metric family.

**Fig. 4.**
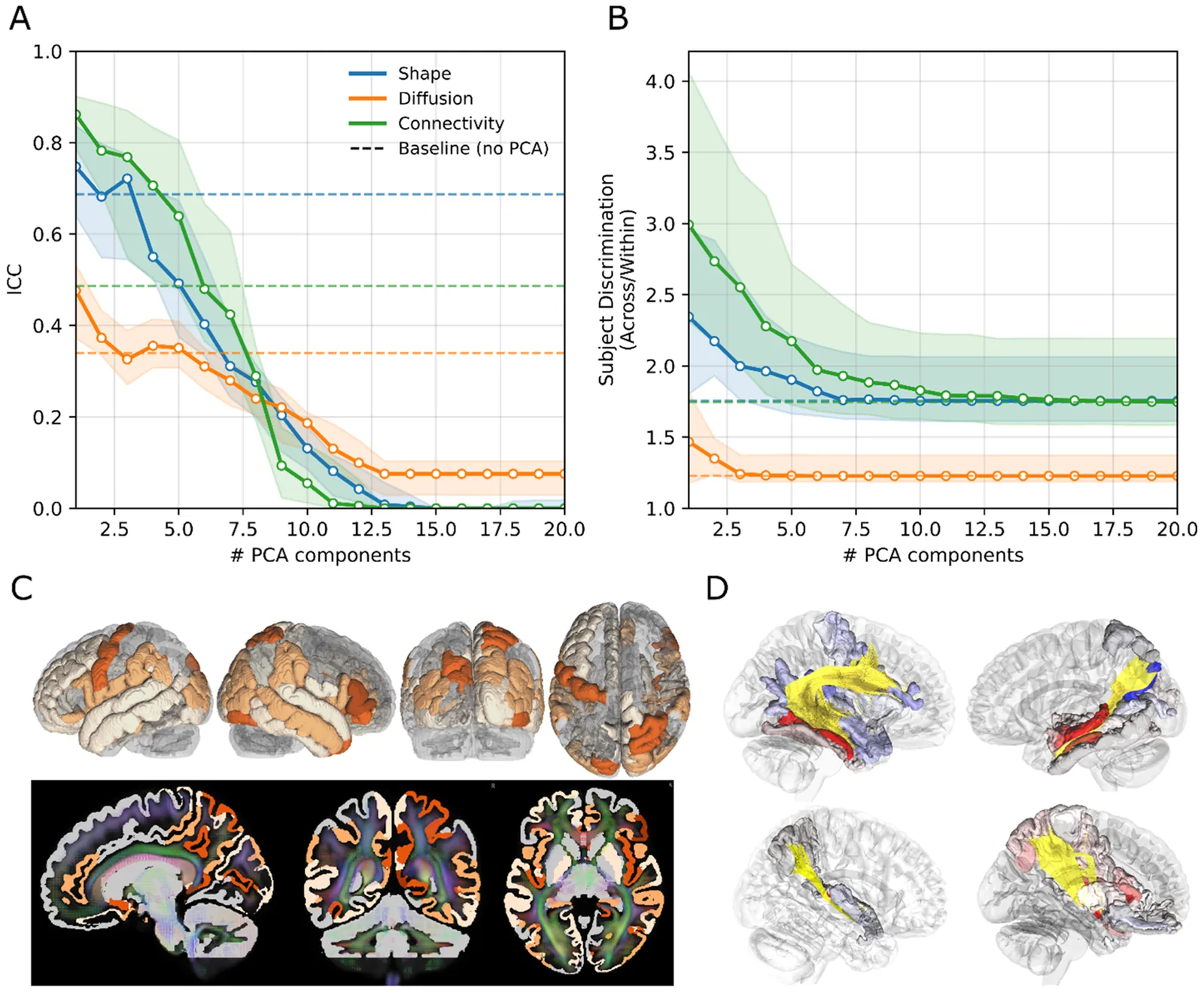
Scanner reproducibility of low-dimensional representations across metric families. (A) Median ICC as a function of retained PCA components for shape (blue), diffusion (orange), and connectivity (green). Dashed lines indicate baseline ICC; shaded regions show interquartile range across 36 tracts. (B) Median subject discrimination as a function of PCA components. (C) Cortical regions contributing to reproducible individual variation in connectivity. Color intensity indicates the number of tracts for which a region is a principal driver (light peach = 1, deep/burnt orange = 4). Bottom row: slices including subcortical structures, overlaid on a QA map with fiber orientation coloring. (D) Example tracts illustrating signed PC1 loading patterns. Warm colors indicate positive loadings, cool colors negative; tract streamlines in yellow.

**Fig. 5.**
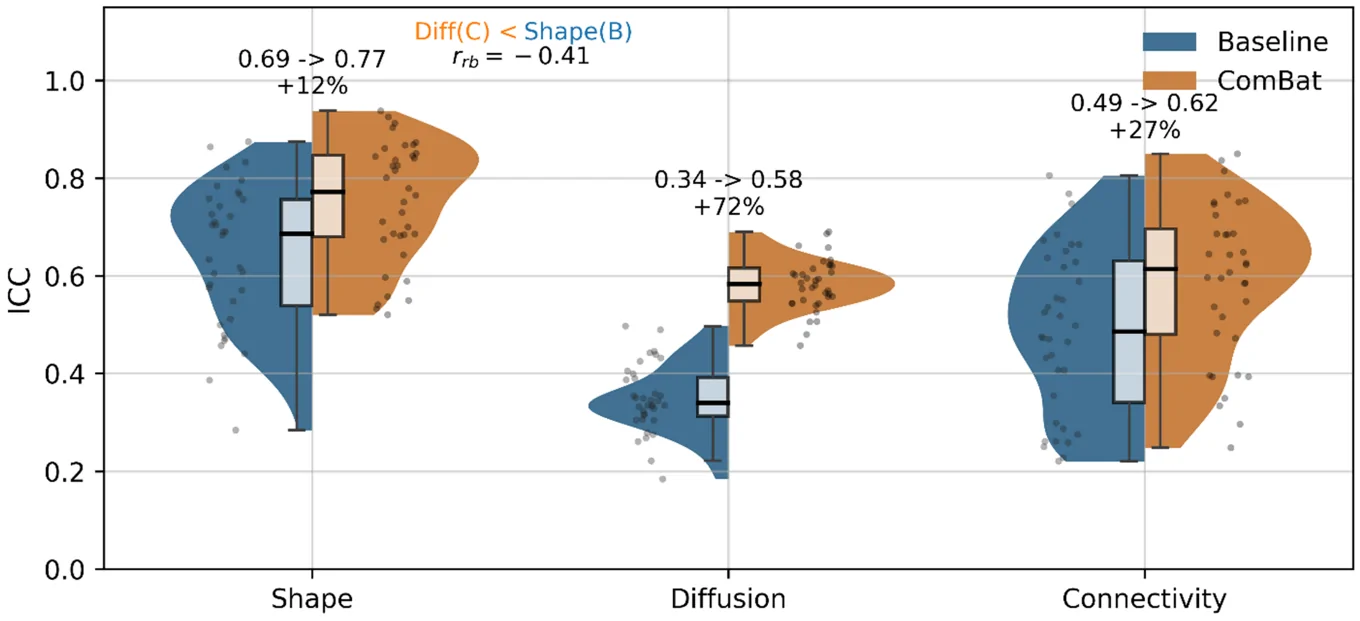
Effect of ComBat harmonization on scanner reproducibility across metric families. Split violin plots showing the distribution of tract-level ICC before (baseline, blue) and after (ComBat, orange) harmonization for shape, diffusion, and connectivity. Individual points represent tract-specific ICC values. Annotations indicate median ICC, percent change, and the rank-biserial correlation (*r*_*rb*_) comparing harmonized diffusion to unharmonized shape.
