# Supplementary material for: Reproducibility of diffusion, shape, and connectivity metrics across scanners: Implications for multi-site tractography
Source: Neuroimage. Author manuscript; Available in PMC 2026 Oct 9. (PMC13651454; doi:10.1016/j.neuroimage.2026.122228)
Supplement: Supplementary file 1 [file NIHMS2213708-supplement-1.docx]

**Supplementary Material**

**Contents**

- Supplementary Table S1. Definitions of shape and connectivity metrics
- Supplementary Figure S1. Tract-level ICC by metric family
- Supplementary Methods. Stability of connectivity PC1 loadings
- Supplementary Results. Stability of connectivity PC1 loadings
- Supplementary Figure S2. Stability of connectivity PC1 loadings
- Supplementary Table S2 (PC1 loadings) and Supplementary Table S3 (stability summary) are provided as separate CSV files; captions below.
- Supplementary Results. ComBat harmonization without subject covariate (sensitivity analysis)
- Supplementary Table S4. Metric-family reliability under alternative ComBat specifications
- Supplementary Figure S3. ComBat harmonization without subject covariate

**Supplementary Table S1. Definitions of shape and connectivity metrics**

*Shape metrics (19 per tract) (Yeh, 2020)*

| **DSI Studio output** | **Figure 2A label** | **Units** | **Description** |
| --- | --- | --- | --- |
| number of tracts | N tracts | count | Number of streamlines forming the reconstructed tract |
| mean length | Mean length | mm | Mean streamline length |
| span | Span | mm | Straight-line distance between the two ends of the tract |
| curl | Curl | unitless | Ratio of length to span; higher values indicate a more curved trajectory |
| elongation | Elongation | unitless | Ratio of length to tract diameter; higher values indicate a longer, thinner bundle |
| total volume | Total vol | mm³ | Volume of voxels occupied by the tract |
| 1st quarter volume | Vol Q1 | mm³ | Volume of the first length-quarter segment |
| 2nd and 3rd quarter volume | Vol Q2-Q3 | mm³ | Volume of the middle two length-quarter segments |
| 4th quarter volume | Vol Q4 | mm³ | Volume of the fourth length-quarter segment |
| total surface area | Surf area | mm² | Surface area of the entire tract |
| total radius of end regions | End radius | mm | Combined radius of the two end (innervation) regions |
| total area of end regions | End area | mm² | Combined surface area of the two end regions |
| irregularity | Irregularity | unitless | Deviation of the tract surface from a smooth cylinder of equal volume |
| area of end region 1 | End1 area | mm² | Surface area of the first end region |
| radius of end region 1 | End1 radius | mm | Radius of the first end region |
| volume of end branches 1 | End1 vol | mm³ | Volume of the branching streamlines at the first end |
| area of end region 2 | End2 area | mm² | Surface area of the second end region |
| radius of end region 2 | End2 radius | mm | Radius of the second end region |
| volume of end branches 2 | End2 vol | mm³ | Volume of the branching streamlines at the second end |

*Connectivity metrics (12–56 per tract) (Yeh, 2022)*

| **Metric** | **Units** | **Description** |
| --- | --- | --- |
| Intersect ratio (per region) | ratio (0–1) | Fraction of a FreeSurferSeg region's voxels overlapped by the tract (track density above a noise floor of 0.5% of maximum). Quantifies the extent of structural connectivity between the tract and the region, interpretable as the relative anatomical footprint of the pathway within that grey-matter region. |

**Supplementary Figure S1. Tract-level ICC by metric family for all 36 association tracts.** Horizontal bar charts showing median ICC (with interquartile range across metrics) for shape (blue), diffusion (orange), and connectivity (green) within each tract. Tracts are sorted by descending shape ICC. N indicates the number of scanning sessions available per tract.

Tract abbreviations: *AF, arcuate fasciculus; Cg, cingulum (subdivisions: FP, frontal-parietal; FPH, frontal-parahippocampal; Olf, parolfactory; PH, parahippocampal; PHP, parahippocampal-parietal; SLF1, superior longitudinal fasciculus I segment); EC, extreme capsule; FAT, frontal aslant tract; HA,*

*hippocampal alveus; IFOF, inferior fronto-occipital fasciculus; ILF, inferior longitudinal fasciculus; MLF, middle longitudinal fasciculus; PAT, parietal aslant tract; SLF2, superior longitudinal fasciculus II; SLF3, superior longitudinal fasciculus III; UF, uncinate fasciculus; VOF, vertical occipital fasciculus. Suffixes -L and -R denote the left and right hemisphere, respectively.*

**Supplementary Methods. Stability of connectivity PC1 loadings**

Because the PCA was estimated and evaluated within the same dataset, we assessed whether the leading connectivity loadings (Supplementary Table S2) reflect stable structure rather than a within-sample artifact, using two resampling schemes. In a leave-one-scanner-out analysis, we refit the connectivity PCA on three of the four scanners, repeating for each held-out scanner (four folds). In a nonparametric bootstrap, we refit on 1000 resamples of the scans drawn with replacement. For each tract and resample, we recomputed the first principal component (PC1) loading vector and compared it to the full-sample vector using Tucker's congruence coefficient, the absolute cosine between the two vectors, which is invariant to the arbitrary sign of an eigenvector (Lorenzo-Seva and ten Berge, 2006); φ ≥ 0.95 indicates near-equivalent loadings.

We additionally quantified retention of the dominant region (whether the largest-magnitude PC1 region matched the full-sample solution) and agreement of the region set accounting for 95% of squared PC1 loadings (the regions in Supplementary Table S2, rendered in Figure 4D). Because this set is small (median 6 regions, range 2–14) and the Jaccard index is sensitive to set size, we report both the Jaccard overlap and recall, the fraction of full-sample regions recovered, which is robust to a resample adding a borderline region. Per-tract stability metrics are provided in Supplementary Table S3.

**Supplementary Results. Stability of connectivity PC1 loadings**

The connectivity PC1 loadings were highly stable under both resampling schemes. Across the 36 association tracts, the median leave-one-scanner-out congruence with the full-sample loadings was φ = 0.99 (IQR 0.98–1.00) and exceeded 0.95 in every tract, indicating that removing any single scanner left the leading loading pattern essentially unchanged. The most strongly weighted region was retained in the majority of folds (mean retention 0.87). Under the more demanding bootstrap, which resamples scans with replacement and therefore perturbs the data more aggressively than removing one scanner, congruence remained high (median φ = 0.96, IQR 0.95–0.98) and met the φ ≥ 0.95 criterion in 27 of 36 tracts. The leading connectivity loading pattern is thus a stable feature of the data rather than an artifact of the particular scanners or scans sampled.

The set of regions defining 95% of the squared PC1 loadings, which is the set we recommend for downstream use and display in Figure 4D, was also largely preserved across resamples. Unlike Tucker's congruence, the Jaccard and recall measures have no established threshold; we therefore interpret them descriptively, treating recall as the primary indicator because it directly reflects whether the regions we report are recovered when the data are resampled. A median 95% of the full-sample regions were recovered in each leave-one-scanner-out fold and 90% in each bootstrap resample (recall). The Jaccard index was lower (median 0.88 leave-one-scanner-out and 0.75 bootstrap) than recall because Jaccard penalizes both omissions and additions, and in tracts with small region sets a resample occasionally promoted one additional borderline region into the 95% set without dropping any full-sample region. The left parietal aslant tract illustrates this: with only two regions in its full-sample set, both were recovered in every fold (recall 1.0), yet the occasional addition of a third region reduced the Jaccard index to 0.58. Because recall is unaffected by such additions, it is the more faithful measure of whether a user following our recommendation would recover the regions we report, and it remained high throughout.

Congruence showed a weak negative association with the number of connected regions per tract (Spearman ρ = −0.31 leave-one-scanner-out, −0.37 bootstrap). This direction is expected on statistical grounds. The connectivity feature space for a tract has as many dimensions as it has connected regions (12 to 56), while the number of scans available to estimate the covariance structure is fixed at 27. Tracts with more regions therefore have a higher ratio of estimated parameters to observations, so their loading vectors, particularly the many small loadings outside the dominant set, are estimated with less precision and are more sensitive to resampling. The effect was nonetheless small in absolute terms, and stability remained high across the full range of region counts: the densest tract retained near-equivalent loadings under leave-one-scanner-out resampling (left inferior fronto-occipital fasciculus, 56 regions, φ = 0.98), while the least congruent tracts were among the sparsest rather than the densest (right cingulum–SLF1, 12 regions, φ = 0.95; right cingulum–parahippocampal, 25 regions, φ = 0.96). The weak dependence on dimensionality thus reflects reduced precision in the long tail of small loadings, consistent with the limitation noted in the main text, rather than instability of the dominant regions on which our recommendation rests.

**Supplementary Figure S2. Stability of connectivity PC1 loadings.** Per-tract medians across 36 association tracts under leave-one-scanner-out (4 folds) and bootstrap (1000 resamples) refitting. (A) PC1 loading congruence with the full-sample solution (Tucker's φ; dashed line, φ = 0.95). (B) Top-95% region-set Jaccard overlap, each tract colored by recall (fraction of full-sample regions recovered). Because that set is small (median 6 regions, range 2–14), Jaccard is conservative: tracts with low Jaccard nonetheless recover their full-sample regions (high recall, lighter markers), the apparent disagreement arising from resamples adding a borderline region rather than dropping a reported one (e.g. left parietal aslant tract, 2 regions, Jaccard ≈ 0.6, recall 1.0; arrow).

**Supplementary Tables provided as separate CSV files**

**Supplementary Table S2** (separate CSV). **PC1 loadings for connectivity metrics across all 36 association tracts.** For each tract, all regions with nonzero overlap, their signed PC1 loading, absolute loading, and rank by absolute loading.

**Supplementary Table S3** (separate CSV). **Stability of connectivity PC1 loadings per tract.** For each of the 36 association tracts: n_conn, number of connected regions; n_top95, number of regions in the 95%-loading set; pc1_congruence_loso_median / _boot_median, median Tucker congruence of the PC1 loadings under leave-one-scanner-out and bootstrap resampling; top1_retention_*, retention of the dominant region; top95_jaccard_* and top95_recall_*, Jaccard overlap and recall (fraction of full-sample regions recovered) of the 95%-loading set. See Supplementary Methods for details.

**Supplementary Results.** ComBat harmonization without subject covariate (sensitivity analysis)

The primary ComBat analysis included subject identity as a biological covariate, which preserves between-subject variance while removing scanner effects. Because this design differs from a conventional multi-site setting, in which different individuals are scanned at each site and subject identity cannot be modelled in this way, retaining subject identity as a covariate may yield optimistic estimates of post-harmonization reliability. To verify that our conclusion does not depend on this modelling choice, we repeated the harmonization using scanner as the only batch variable, with no subject covariate, which corresponds to the conventional multi-site setting.

Post-harmonization estimates were somewhat lower without the covariate, as expected: median ICC was 0.73 for shape, 0.53 for diffusion, and 0.56 for connectivity, compared with 0.77, 0.58, and 0.62 respectively with the covariate (Supplementary Table S4, Supplementary Figure S3). The central finding was unchanged. Harmonized diffusion (0.53) remained well below unharmonized shape (0.69), and the effect size for this comparison increased rather than decreased (rank-biserial correlation −0.66, compared with −0.41 in the covariate-adjusted analysis). The cross-family ordering, and the conclusion that metric family selection remains consequential after harmonization, therefore holds, and is if anything more pronounced, under the more realistic harmonization setting.

**Supplementary Table S4. Metric-family reliability under alternative ComBat specifications**

| **Metric family** | **Baseline ICC** | **ComBat, with subject covariate** | **ComBat, scanner only (no subject covariate)** |
| --- | --- | --- | --- |
| Shape | 0.69* | 0.77 (+12%) | 0.73 (+7%) |
| Diffusion | 0.34 | 0.58 (+72%)* | 0.53 (+57%)* |
| Connectivity | 0.49 | 0.62 (+27%) | 0.56 (+16%) |

*Harmonized diffusion vs. unharmonized shape: rank-biserial correlation −0.41 (with subject covariate) and −0.66 (scanner only). Values are medians across 36 association tracts.*

**Supplementary Figure S3**. **ComBat harmonization without subject covariate**. Split violin plots of tract-level ICC before (baseline, blue) and after (ComBat, orange) harmonization for each metric family, with ComBat applied using scanner as the only batch variable and no subject covariate, corresponding to a conventional multi-site setting. Annotations give median ICC, percent change, and the rank-biserial correlation comparing harmonized diffusion to unharmonized shape. Harmonized diffusion (0.53) remains below unharmonized shape (0.69), and the cross-family hierarchy is preserved.
